# Uncovering the tacit: a qualitative study of obstetric nurses’ risk recognition and decision-making in perinatal care

**DOI:** 10.3389/fmed.2025.1701924

**Published:** 2025-12-19

**Authors:** Shibo Zhang, Yi Chen, Lu Hua, Yang He, Liu Zhang

**Affiliations:** Chongqing Health Center for Women and Children, Chongqing, China

**Keywords:** obstetric nursing, clinical risk recognition, decision-making, clinical judgment, Tanner model, reflexive thematic analysis

## Abstract

**Aim:**

This study explored how obstetric nurses identify and manage clinical risks under high-pressure and uncertain conditions, with emphasis on the tacit cognitive processes shaping their judgment.

**Design:**

A qualitative descriptive study was conducted using a phenomenological approach, based on Tanner’s Clinical Judgment Model.

**Methods:**

Semi-structured, in-depth interviews were conducted with 25 obstetric nurses from three tertiary hospitals in western China. Data were analyzed using reflexive thematic analysis following Braun and Clarke’s six-phase approach. The interview guide was developed and refined according to Tanner’s four phases—noticing, interpreting, responding, and reflecting—to capture the multidimensional nature of clinical judgment.

**Results:**

A total of 11 interconnected subthemes were identified across the four dimensions. In the noticing phase, nurses recognized baseline conditions, detected atypical cues, and developed experience-driven vigilance. The interpreting phase involved synthesizing multi-source cues and, critically, managing conflicting signals when subjective impressions and objective indicators diverged—a key finding that reveals how intuitive and analytical reasoning interact in clinical ambiguity. The responding phase encompassed prioritizing action, communicating and seeking peer confirmation, and acting under uncertainty. The reflecting phase entailed recurrent self-review, reflective learning, and the development of heightened risk sensitivity. A cross-cutting theme of sustained risk vigilance permeated all stages, representing nurses’ enduring attentiveness to subtle warning signs and potential deterioration.

**Conclusion:**

Obstetric risk recognition is not merely technical but an iterative cognitive process integrating perception, experience, emotion, and reflection. Nurses’ risk vigilance functions as a tacit safeguard in managing uncertainty and ensuring maternal safety.

**Clinical implications:**

The findings highlight the need to strengthen a simulation-based training program that targets ambiguous cues, promote reflective debriefing and peer learning, and design institutional environments that support collaborative and emotionally sustainable decision-making in obstetric care.

## 1 Introduction

As healthcare increasingly transitions from exclusively hospital-based settings to encompass community and home environments, nurses are playing an ever more critical role in the early identification of clinical risks. In obstetric care, nurses occupy frontline positions in the maternal–infant safety continuum, serving as the primary perceptual gatekeepers for early warning signs (1). Globally, maternal mortality and severe perinatal complications remain pressing public health issues. The MBRRACE-UK report (2) indicated that more than 50% of preventable maternal deaths were associated with delays in recognizing or responding to clinical deterioration. Similarly, Baird and Graves (3) reported that early warning signs were present but unrecognized or unaddressed in 39% of severe perinatal incidents. Comparable challenges have also been observed in Chinese obstetric settings, where the national maternal mortality ratio (MMR) was approximately 15.7 per 100,000 live births in 2022 (4). Despite significant progress in reducing overall maternal deaths, preventable causes—often linked to delayed recognition and response to clinical deterioration—remain a major concern in hospital-based obstetric care.

Despite the widespread implementation of standardized early warning systems such as the Modified Early Obstetric Warning System (MEOWS), current approaches to obstetric risk recognition remain predominantly tool-driven. These instruments demonstrate high sensitivity (median 89%) and specificity (median 85%) in predicting severe complications and intensive care unit (ICU) admissions; however, their positive predictive values are limited, leading to frequent false alarms and potential alarm fatigue (5). More importantly, such systems primarily capture physiological deviations—blood pressure, temperature, and heart rate—while overlooking the complex and often ambiguous cues that nurses encounter in real-world clinical environments.

There is limited understanding of how nurses actually recognize and interpret risks in practice. Existing literature focuses largely on compliance with risk tools and institutional protocols, providing little insight into the cognitive, experiential, and emotional dimensions of nurses’ judgment during high-pressure obstetric care. As Smith et al. highlighted in a systematic review, “there is little evidence regarding how nurses’ decision-making interacts with these tools in practice,” revealing a significant gap in our understanding of the human cognitive processes underlying risk recognition (6). In high-pressure obstetric contexts—such as preeclampsia, fetal distress, or postpartum hemorrhage—conditions can progress rapidly, rendering structured early warning tools insufficient for timely detection (7). When clinical manifestations are atypical or fluctuate within short timeframes, nurses’ subjective judgments become crucial in determining the appropriate timing for intervention (8). However, most studies continue to emphasize the technical performance of risk tools and institutional compliance, while overlooking nurses’ experiential knowledge and cognitive pathways as active agents in risk recognition (9).

While existing qualitative studies have examined aspects of clinical judgment, the majority of them have conceptualized judgment as either a sequential cognitive process or a tool-driven task, offering limited insight into the tacit, emotionally mediated, and context-bound reasoning nurses use in fast-evolving obstetric scenarios (10). Building on this literature, the present study provides two key extensions. First, it offers an empirically grounded elaboration of Tanner’s model by illustrating how noticing, interpreting, responding, and reflecting unfold in a fluid and overlapping manner, shaped by ambiguity, shifting cues, and emotional appraisal. Second, it contributes a culturally embedded perspective by demonstrating how relational expectations, hierarchical dynamics, and family involvement within Chinese obstetric care influence nurses’ judgment processes. These theoretical and contextual insights advance our current understanding of how frontline nurses make decisions in environments where risk is often uncertain, dynamic, and not fully captured by standardized early-warning tools (11).

This study addresses that gap by moving beyond standardized measurement to explore the tacit processes through which obstetric nurses detect, interpret, and respond to evolving clinical risks. Based on Tanner’s Clinical Judgment Model (12), it elucidates how nurses’ noticing, interpreting, responding, and reflecting unfold dynamically under conditions of uncertainty. By uncovering these cognitive pathways, the study contributes empirical and theoretical insights for strengthening clinical judgment training and enhancing obstetric risk governance. Therefore, this study aimed to explore how obstetric nurses recognize, interpret, and respond to evolving clinical risks under conditions of uncertainty, with particular attention to the tacit cognitive and experiential processes shaping their judgment.

## 2 Methods

### 2.1 Aim

This study aimed to explore the judgment mechanisms and experiential strategies used by obstetric nurses in identifying, interpreting, and responding to clinical risks. It further aimed to uncover their perceptual logic, decision-making processes, and behavioral patterns within complex clinical environments.

### 2.2 Design

A qualitative exploratory design was adopted, based on interpretivist and constructivist paradigms that emphasize the situated and subjective nature of experience (13). Clinical judgment was conceptualized as an embedded practice, and in-depth interviews were conducted to capture nurses’ lived experiences in risk recognition. Data analysis was performed based on Tanner’s Clinical Judgment Model (12), in combination with theoretical developments in nursing cognition and decision-making, to guide theme generation and interpretation.

### 2.3 Setting and participants

The study was conducted in the obstetric units of three tertiary-level hospitals in western China, including general obstetric wards, high-risk obstetric wards, and mother–infant rooming–in units. A purposive maximum variation sampling strategy was used. The inclusion criteria were as follows: (1) registered nurse qualification, (2) at least 2 years of frontline obstetric clinical experience, and (3) currently engaged in routine patient care. Nurses in administrative positions, interns, or those within 1 year postpartum were excluded from the study. A total of 25 nurses were recruited, aged 24–44 years, with 2–21 years of work experience. Their professional ranks ranged from junior, associate, and senior to and chief levels, representing diverse educational backgrounds, roles, shifts, and gender diversity.

### 2.4 Ethical considerations

This study was conducted in accordance with ethical guidelines for research involving human participants and received approval from the relevant ethics committee in July 2025 (2025 Lun Shen (Ke) 050). All participants provided written informed consent prior to the interviews, confirming their voluntary participation and their right to withdraw at any time without adverse consequences. The research team ensured participant anonymity, and all data were used solely for research purposes and stored on encrypted devices accessible only to authorized personnel, adhering to the principles of the Declaration of Helsinki.

### 2.5 Recruitment

Participants were recruited through multiple channels, including coordination by hospital nursing departments, WeChat announcements, peer referrals, and recommendations from head nurses. Researchers provided one-on-one explanations of the study, data handling procedures, and participants’ rights to ensure informed consent. To minimize hierarchical influence, head nurses and managers did not participate in the screening or interview processes.

Of the 30 nurses initially approached, 25 agreed to participate and completed interviews. In total, five declined due to scheduling conflicts or personal reasons, and no further demographic information was collected to protect anonymity. The characteristics of those who declined did not appear to differ systematically from those of the participants in terms of roles or years of experience.

### 2.6 Data collection

Data were collected through semi-structured, in-depth one-on-one interviews. The interview guide was developed based on Tanner’s Four-Stage Clinical Judgment Model (12) and was iteratively refined through literature review, expert consultation, and pilot feedback. Sample questions included: “How do you recognize subtle but unusual changes in a postpartum woman’s condition?” “When clinical indicators appear normal but you sense potential risk, what actions do you typically take?,” and “Have you ever repeatedly reflected on a clinical decision that may have been a misjudgment and found it affected your later practice?” (Supplementary material 1). All interviews were conducted in a quiet and private setting and lasted between 35 and 75 min. They were audio-recorded with consent and then transcribed verbatim by a trained research assistant. Theme saturation was assessed iteratively following Saunders’ guidance (14). During concurrent data collection and analysis, the team monitored three criteria: (1) the absence of new codes in the coding matrix, (2) increasing redundancy across data excerpts, and (3) the stability of the emerging thematic structure across participants. After the 22nd interview, no additional concepts were identified, and subsequent transcripts largely reinforced existing patterns. Three more interviews were conducted to confirm this stability. All analysts reviewed the coding output and thematic memos independently and then reached consensus through discussion that saturation had been achieved, ensuring that the final themes were conceptually comprehensive and sufficiently representative of the dataset.

All interviews were conducted by two members of the research team with formal training in qualitative interviewing. The first author, a senior obstetric nurse with over 10 years of frontline experience, conducted 18 interviews, and the second analyst, a nursing education researcher with qualitative expertise, conducted the remaining 7 interviews. Both interviewers had received training in reflexive interviewing and met regularly to discuss interview dynamics, ensure consistency in probing style, and reflect on how their professional backgrounds might shape data collection. This approach strengthened credibility and reflexivity throughout the data-gathering process.

### 2.7 Coding and data analysis

Data were analyzed using reflexive thematic analysis, as described by Braun and Clarke (15). The process involved six steps: (1) data familiarization, (2) open coding, (3) theme generation, (4) theme review and refinement, (5) theme naming and definition, and (6) report writing. Two researchers (SZ and XL) independently coded the transcripts using NVivo 12 Plus. Coding discrepancies were discussed in regular team meetings until full consensus was achieved, supported by iterative comparison with the raw data and codebook refinement. Although inter-rater reliability was not statistically calculated, agreement was reached through reflexive dialog and collaborative validation, consistent with the interpretivist paradigm of reflexive thematic analysis.

The first author led the initial inductive coding and theme development, while the second researcher conducted parallel coding for verification. The third author reviewed all codes and thematic maps, ensuring coherence between data excerpts and analytic interpretations. An external qualitative expert further audited the coding framework to enhance dependability and confirmability. Final theme structures were established through consensus after extensive comparison with the raw data.

Throughout the analysis, the team actively searched for negative cases and disconfirming evidence to test the robustness of emerging interpretations. Instances that diverged from dominant patterns—such as nurses who primarily relied on protocol-driven reasoning rather than experiential cues, or cases in which subjective discomfort did not prompt heightened vigilance—were examined in depth. These deviations informed the refinement of theme boundaries and clarified the conditions under which certain patterns did or did not apply, strengthening confirmability and analytic rigor.

### 2.8 Reflexivity

The research team maintained an explicit stance of reflexivity throughout the study to minimize bias and enhance interpretive transparency. The two lead analysts combined more than 10 years of experience in obstetric nursing and qualitative inquiry—one as a senior obstetric nurse with extensive frontline experience in high-risk maternity care and the other as a nursing education researcher specializing in professional cognition and clinical judgment.

This dual background provided both insider and outsider perspectives: the clinician offered contextual sensitivity to subtle obstetric cues and workplace realities, while the educator contributed theoretical distance and critical questioning of implicit assumptions. Recognizing that professional experience could shape data interpretation, the researchers engaged in continuous reflexive dialog to bracket preconceptions about “good practice” or “ideal decision-making.”

Reflexive strategies included maintaining analytic memos, documenting assumptions before and during analysis, and regularly revisiting how personal values, emotional reactions, and disciplinary orientations might influence theme development. Independent coding, peer debriefing, and external audit further ensured that emergent interpretations remained grounded in participants’ narratives rather than researchers’ expectations.

### 2.9 Trustworthiness

To ensure the trustworthiness and scientific rigor of the findings, the study adhered to Lincoln and Guba’s criteria for naturalistic inquiry, including credibility, confirmability, dependability, and transferability (16). Credibility was enhanced through independent coding by two researchers, collaborative discussions, and member checking with participants. Confirmability was supported by maintaining comprehensive research logs, coding decision records, and reflexive memos, ensuring transparency and traceability. Dependability was reinforced by clearly documenting the research procedures, including participant recruitment, interview guide development, saturation assessment, and thematic analysis strategy. Transferability was achieved through detailed descriptions of participants, data collection environments, and clinical contexts, enabling applicability in similar nursing settings.

For member checking, five participants were purposively selected to represent variation in years of experience (junior to senior levels) and clinical settings (general, high-risk, and rooming-in wards). Each participant reviewed a concise summary of the preliminary themes and confirmed that the interpretations accurately reflected their experiences.

An external audit was conducted by a qualitative research expert who was not involved in data collection or preliminary coding. The auditor reviewed the full codebook, sample-coded transcripts, thematic maps, and analytic memos to evaluate the coherence between data excerpts and emerging interpretations. Feedback focused on refining theme boundaries, strengthening the distinction between overlapping subthemes, and ensuring that interpretations remained grounded in participants’ narratives. The research team incorporated these suggestions through iterative discussion and theme revision. This constituted a formal analytic audit rather than an informal peer consultation, enhancing dependability and confirmability.

### 2.10 Participant characteristics

A total of 25 obstetric nurses participated in this study, with their demographic characteristics summarized in Table 1. The majority of the nurses were female (*n* = 22), with ages ranging from 24 to 44 years. The majority of the participants had more than 3 years of clinical experience in obstetric care (*n* = 21). In terms of educational background, 9 participants held postgraduate degrees and 16 held bachelor’s degrees.

**Table 1:** Demographic characteristics of obstetric nurse participants.

ID	Sex	Age	Education	Title	Years_OB	Clinical setting
N1	Female	38	Bachelor	Senior nurse	8	High-risk ward
N2	Male	34	Bachelor	Senior nurse	8	General ward
N3	Female	43	Bachelor	Associate chief nurse	21	High-risk ward
N4	Female	39	Bachelor	Senior nurse	14	Rooming-in unit
N5	Female	31	Bachelor	Nurse	7	General ward
N6	Female	26	Postgraduate	Nurse	1	General ward
N7	Female	36	Bachelor	Senior nurse	8	High-risk ward
N8	Male	36	Bachelor	Senior nurse	14	General ward
N9	Female	28	Postgraduate	Nurse	3	Rooming-in unit
N10	Female	31	Bachelor	Nurse	5	General ward
N11	Female	24	Bachelor	Nurse	2	General ward
N12	Female	40	Bachelor	Senior nurse	17	High-risk ward
N13	Female	33	Postgraduate	Senior nurse	8	General ward
N14	Female	28	Bachelor	Nurse	8	Rooming-in unit
N15	Female	29	Bachelor	Nurse	8	General ward
N16	Female	36	Bachelor	Senior nurse	12	High-risk ward
N17	Male	38	Bachelor	Senior nurse	14	General ward
N18	Female	27	Bachelor	Nurse	2	Rooming-in unit
N19	Female	41	Bachelor	Senior nurse	18	High-risk ward
N20	Female	35	Postgraduate	Nurse	6	General ward
N21	Female	30	Bachelor	Nurse	5	General ward
N22	Female	44	Bachelor	Associate chief nurse	20	High-risk ward
N23	Male	32	Bachelor	Nurse	9	Rooming-in unit
N24	Female	27	Postgraduate	Nurse	2	General ward
N25	Female	37	Bachelor	Senior nurse	13	High-risk ward

All participants were full-time staff nurses at the time of data collection.

## 3 Findings

**Figure 1:**
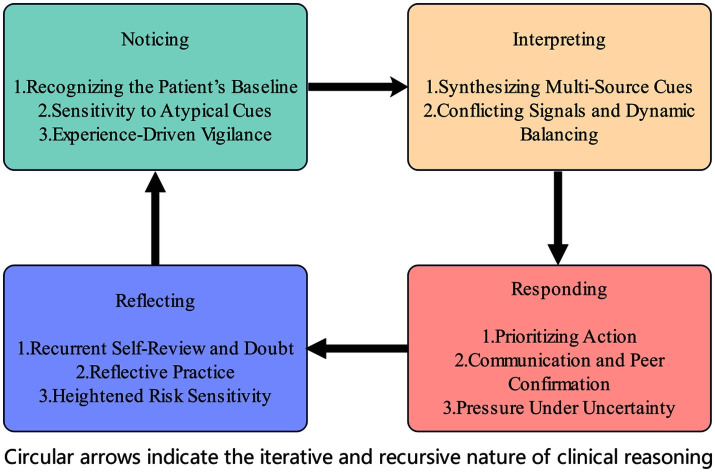
Presentation of the results.

Thematic analysis generated a framework of four dimensions—noticing, interpreting, responding, and reflecting—comprising 11 interconnected subthemes (Supplementary material 2). In the noticing phase, nurses recognized patients’ baseline states, remained sensitive to atypical cues, and exercised experience-driven vigilance. The interpreting phase involved synthesizing multi-source cues and balancing conflicting signals. The responding phase encompassed prioritizing action, engaging in communication and peer confirmation, and managing pressure under uncertainty. The reflecting phase highlighted recurrent self-review and doubt, reflective practice, and heightened risk sensitivity. Underpinning all dimensions was a cross-cutting theme of sustained “risk vigilance” toward maternal safety (Figure 1), which functioned as a continuous thread linking perception, reasoning, and action across phases, reinforcing nurses’ proactive stance toward maternal safety (Supplementary material 1).

### 3.1 Noticing

In obstetric care, nurses must rely on keen observation to detect changes in a patient’s condition. These subtle changes often serve as early indicators of potential clinical deterioration, making nurses’ observational acuity critical to risk identification.

#### 3.1.1 Recognizing the patient’s baseline

Through sustained observation of maternal physiology, behavior, and emotional state, nurses develop a mental model of each patient’s baseline. This familiarity allows them to detect even minor deviations. Nurses frequently rely on patterns in vital signs such as temperature, blood pressure, and heart rate to establish what is “normal” for a specific patient.

“The patient’s temperature is usually around 36.5 °C, but at that moment it had risen slightly to 37.0 °C.” (N3)

Behavioral and emotional patterns are equally important in forming this baseline.

“She’s usually very active, likes walking around and chatting with others.” (N20)

“She used to sleep well every night, but recently she’s had insomnia for three consecutive days, wandering the ward at 2 a. m.” (N2)

#### 3.1.2 Sensitivity to atypical cues

Nurses must remain alert to atypical cues that may not conform to standard pathological signs but still suggest risk. These include behavioral withdrawal, emotional shifts, or psychosocial changes that are not immediately reflected in physiological data.

“Today she was unusually silent and reluctant to speak, which made me suspect postpartum depression.” (N12)

“Her voice became faint, she avoided eye contact, and appeared exhausted—this was not her normal demeanor.” (N19)

Early signs can also manifest through subtle facial expressions or body language.

“There were no obvious expressions of discomfort, but I noticed her lips were slightly pale and tense. I decided to check her blood glucose for possible hypoglycemia.” (N17)

Changes in how the patient interacts with family, roommates, or staff can also serve as atypical risk indicators.

“She usually asks a lot of questions and chats with her neighbor about childcare. But that day she said nothing, didn’t respond when called. I became very alert—and later she had an emotional breakdown at night.” (N4)

#### 3.1.3 Experience-driven vigilance

With years of clinical exposure, nurses develop an intuitive sensitivity to risk, even in the absence of clear physiological abnormalities.

“Based on my experience with mastitis cases, when I saw her frown during breastfeeding and noticed localized redness, I immediately recommended a breast exam.” (N11)

Experienced nurses are often attuned to temporal cues—when recovery is slower or faster than expected.

“It was three days after her C-section, and she still said the incision hurt badly when walking. That raised a red flag for me—normally, patients can get out of bed by then. I asked the doctor to reassess the wound.” (N1)

Triggers for vigilance may also arise from contextual cues unrelated to the patient’s body—such as staffing shortages, environmental shifts, or unusual family behavior.

“We were short-staffed today, and two high-risk patients had just been admitted. I knew I had to increase my rounds to prevent missing anything.” (N8)

“Her husband is usually quiet, but today he kept asking questions and even peeked at her chart. I checked on her immediately—she was pale, so I initiated close monitoring.” (N21)

### 3.2 Interpreting

In this phase, nurses synthesize diverse clinical cues—physiological, psychological, emotional, and behavioral—to develop a holistic risk assessment and support timely decision-making.

#### 3.2.1 Synthesizing multi-source cues

By integrating data from multiple sources, nurses build more accurate clinical inferences.

“Her blood pressure rose slightly to 140/90 mmHg, and she kept rubbing her temples and said her vision was blurry. Considering her history of gestational hypertension and that it was day three postpartum, I suspected postpartum hypertension syndrome.” (N13)

This integrative approach is especially important when no single indicator clearly signals deterioration.

“She seemed quiet and cooperative, but I noticed her pulse was slightly elevated and her face was pale. Given her history of mild anemia, I suspected this might be symptomatic.” (N22)

#### 3.2.2 Conflicting signals and dynamic balancing

Sometimes, patient cues conflict—particularly when subjective complaints and objective signs do not align—placing higher demands on nurses’ reasoning and synthesis abilities.

“Her heart rate and blood pressure were completely normal, but she kept pressing the call bell, saying she felt tightness in her chest.” (N5)

In such cases, nurses avoid relying on any single metric. Instead, they triangulate verbal reports, behavior, emotional state, and medical history.

“She repeatedly told me she felt like she was going to collapse, but all her vitals were within range. After talking to her family, I suspected it was a stress response.” (N16)

Contradictory cues require careful interpretation. Nurses try to identify psychological factors when physical explanations are insufficient.

“She complained of severe breast pain, but her breasts were soft, and milk was flowing well. By observing her posture during breastfeeding, I realized she was psychologically distressed—her pain was more emotional than physical.” (N25)

Rather than rushing to judgment, nurses adopt a “monitor-reassess-respond” cycle, dynamically adjusting their assessment.

“I didn’t make an immediate decision. I watched her for a few hours to see whether her complaints persisted or new symptoms developed before calling the doctor.” (N12)

### 3.3 Responding

Once a potential risk is identified, nurses must make rapid and well-prioritized decisions. The responding phase involves not only prompt intervention but also collaborative judgment and resource management in conditions often marked by unclear and incomplete information.

#### 3.3.1 Prioritizing action

Upon recognizing a clinical threat, nurses assess the urgency and severity to determine and implement the most immediate and effective intervention.

“The patient suddenly experienced heavy vaginal bleeding and a sharp drop in blood pressure. I immediately established an IV line for rapid fluid resuscitation, called the physician, and prepared emergency medications to stabilize her vital signs.” (N15)

When multiple issues occur simultaneously, nurses must quickly distinguish between problems requiring immediate attention and those that can be deferred.

“The patient had a fever, breast engorgement, and emotional distress. I first managed the fever with physical cooling and ordered labs for infection markers, then addressed the breast issue and offered psychological support.” (N24)

#### 3.3.2 Communication and peer confirmation

In complex or ambiguous situations, effective communication with colleagues, physicians, or other team members is essential for cross-verification and clinical accuracy.

“I reported the situation to the on-duty doctor and discussed it with a senior nurse.” (N10)

When uncertain, nurses actively seek second opinions from their peers to reduce the risk of misjudgment.

“When I’m unsure, I always ask another colleague to assess the patient as well.” (N25)

#### 3.3.3 Pressure under uncertainty

When risk signals are vague or evidence is insufficient, nurses still need to act, often bearing significant psychological pressure in making these high-stakes decisions.

“Sometimes we’re really not sure—like when a slight fever might or might not mean an infection. If we report it, it leads to lab tests, hospitalization, and stress for the family. But if we don’t, and something goes wrong, I’d be consumed with guilt. I spend the whole night worrying.” (N2)[SIC]

In such cases, nurses often feel a moral imperative to “do something,” even at the cost of potential overreaction.

“It was 2 a. m., and the patient said she felt dizzy, though all readings were normal. I still called the doctor. Later, they said it was nothing serious, but I don’t regret it—missing something could’ve had serious consequences.” (N6)

### 3.4 Reflecting

Reflection is central to nurses’ professional growth and clinical refinement. By revisiting and questioning their decisions, nurses identify areas for improvement and develop more adaptive strategies for future care. Reflection is not only an emotional regulation process but also a critical mechanism for enhancing care quality in complex settings.

#### 3.4.1 Recurrent self-review and doubt

After unexpected events, nurses often revisit their clinical decisions, wondering if earlier or different actions could have yielded better outcomes.

“I once managed a case of postpartum hemorrhage. Although the patient stabilized in the end, I still wonder whether I should’ve responded faster or called the doctor earlier. It leaves me uneasy.” (N9)

Such reflection helps recalibrate nurses’ internal standards and enhances decisiveness in future high-stakes scenarios.

“When a patient had abdominal pain, I waited half an hour before ordering an ultrasound. Nothing critical was missed, but I kept questioning whether I should have acted sooner. Next time, I definitely will.” (N14)

#### 3.4.2 Reflective practice

After completing a clinical episode, many nurses systematically review the care process to evaluate successes and shortcomings.

“After managing a case of postpartum depression, I realized I had been too focused on physiological indicators and overlooked the patient’s emotional cues.” (N1)

Reflective practice allows nurses to convert experience into knowledge and improve their decision-making and responsiveness over time.

“Following a case of postpartum complications, I started documenting all my interventions and sharing them during our weekly team meetings. Through group discussions, I realized I could have assessed risk faster.” (N17)

Over time, nurses’ accumulated reflections enhance their ability to anticipate risk and implement preventive strategies proactively.

“Through past reflections, I found that first-time mothers often show signs of severe parenting anxiety around day three postpartum. Now I start monitoring their mental status from day two.” (N7)

#### 3.4.3 Heightened risk sensitivity

After encountering serious or near-miss events, nurses often develop heightened sensitivity to similar warning signs. These reflexive responses, though not always grounded in objective logic, strengthen defensive practices and reflect experiential learning.

“Since I handled a case of severe postpartum hemorrhage, I’ve become hypervigilant. If a patient shows even slight discomfort, I immediately order a blood test. Sometimes results are normal, but I’d rather be safe than sorry.” (N13)

In uncertain scenarios, nurses often prefer to act preemptively—even if it leads to over-intervention—because the cost of inaction feels unacceptable.

“She only mentioned slight dizziness, and her blood pressure was fine. But I still had her lie down, gave fluids, and checked her glucose. I didn’t want to repeat the mistake from a previous case.” (N8)

## 4 Discussion

Our findings support and extend Tanner’s Clinical Judgment Model (12). Nurses’ reliance on experiential knowledge during noticing, their iterative synthesis of cues in interpreting, and their rapid prioritization of actions in responding align closely with Tanner’s original stages. At the same time, the data reveal features that stretch beyond the model’s linear structure. In practice, noticing, interpreting, and responding often occur simultaneously, shaped by fluctuating cues, time pressure, and emotional appraisal. Emotional processes such as anticipatory concern, embodied vigilance, and the moral weight of uncertainty functioned as cross-cutting influences across all phases—elements not explicitly addressed in Tanner’s framework. These patterns emerged inductively from participants’ narratives and should therefore be understood as empirically derived observations rather than predetermined theoretical constructs. Based on these insights, we propose that “emotionally engaged reasoning” and “risk vigilance” may represent data-driven refinements to Tanner’s model by highlighting its more cyclical, dynamic, and relational nature.

While these findings resonate with international research (17, 18), they remain shaped by China’s organizational and sociocultural context. Features such as heightened sensitivity to hierarchical expectations and family involvement reflect locally embedded reasoning processes rather than universal patterns. Thus, any broader generalization should be made cautiously and supported by comparative evidence across diverse healthcare systems.

First, in the “noticing” phase, nurses demonstrated heightened sensitivity to deviations from a personalized baseline of the patient’s normal state—particularly in behavior, speech, and emotional cues. This “baseline shift as risk” pattern aligns with the emerging concept of contextual noticing, which emphasizes how accumulated experience sharpens nurses’ ability to detect subtle departures from the norm (19). Some participants also interpreted family members’ unusual anxiety or repeated questioning as early warning signs, illustrating socially distributed risk awareness. This raises ethical issues regarding family participation, confidentiality, and responsibility in Chinese maternity care (20).

Clinical judgment was not linear. Under ambiguous or conflicting signals—such as normal vital signs despite subjective discomfort—nurses engaged in an iterative “sense-verify-re-evaluate” cycle (21). This recursive reasoning mirrors the “spiral of thinking” (22) and emphasizes contextual responsiveness. The ability to reconcile “conflicting signals” emerged as a central finding, reflecting advanced interpretive competence and aligning with recent work on uncertainty management in nursing (23, 24).

Intuition also played a crucial role. Rather than being irrational, it reflected expert pattern recognition and embodied experience (25, 26). Nurses often “acted first, verified later,” demonstrating anticipatory reasoning consistent with the recognition-primed decision model (27, 28). This supports current literature recognizing intuition as a disciplined cognitive process rather than instinct (29).

Moreover, reflection emerged not as a *post-hoc* add-on but as a central mechanism shaping clinical judgment. Participants engaged in a systematic self-review, often questioning their decisions and internalizing the emotional impact of “near misses” to inform future vigilance. Such reflection-in-action was instrumental in enhancing situational awareness and risk perception (30), allowing nurses to build more forward-looking, experience-based frameworks for clinical assessment. However, several nurses described persistent anxiety and hypervigilance after adverse events, suggesting secondary traumatic stress that gradually shaped their future responses. This underscores the need for structured psychological support and post-event debriefing systems to maintain professional resilience (31, 32).

Importantly, our study highlights the often-overlooked role of emotion in frontline decision-making. During night shifts, under staffing shortages, or when facing ambiguous risk signals, nurses frequently exhibited a defensive decision-making style—“erring on the side of action”—driven by acute awareness of responsibility and potential consequences. This behavioral logic reflects not a flaw in procedural judgment but a rational prioritization of psychological safety in high-stakes environments. Nurses willingly accepted the risk of being “overcautious” to avoid the burden of delayed intervention. These findings resonate with research in emergency care (33), which concluded that emotions are not distractors but essential catalysts in nurses’ action initiation. At the same time, this pattern reflects broader systemic pressures, including hierarchical accountability and limited staffing, which often push nurses toward defensive or precautionary actions (34, 35). Addressing these structural influences through supportive leadership and fair risk governance could help balance safety with professional autonomy.

Taken together, our findings suggest that risk recognition training should move beyond mere mastery of alert tools. Training programs should incorporate modules on recognizing ambiguous cues (36), practicing reflective reasoning (37), and conducting team-based debriefings (38). Simulation exercises should emphasize uncertain or atypical cases rather than standardized scenarios to strengthen nurses’ preparedness for real-world complexity (11). In parallel, healthcare management must acknowledge the emotional burden of risk judgment and provide supportive environments that encourage collaborative, not solitary, decision-making (39). Future educational models could also integrate emotional regulation and ethical reasoning components to foster resilience and moral clarity under uncertainty (40).

While parallels can be drawn with international research, contextual differences across healthcare systems must also be acknowledged. In countries with lower nurse-to-patient ratios and greater professional autonomy—such as Australia or Scandinavia—obstetric nurses often exercise more independent judgment and shared accountability within multidisciplinary teams (41, 42). In contrast, within the Chinese healthcare system, heavy workloads, hierarchical structures, and limited staffing create distinct cognitive and moral pressures that shape decision-making. These structural factors may intensify defensive reasoning and constrain collaborative reflection, underscoring the need to interpret clinical judgment within diverse healthcare contexts.

These findings should also be interpreted within the sociocultural framework of Chinese healthcare. The collectivist orientation emphasizes relational harmony, family involvement, and respect for hierarchy, which influence nurses’ moral reasoning and sense of accountability (43). Strong hierarchical relationships and physician dominance can limit nurses’ autonomy while heightening their responsibility for outcomes. This dual dynamic—limited authority yet high accountability—shapes a cautious, emotionally engaged approach to obstetric risk recognition, reflecting culturally embedded reasoning in practice (44). While these patterns resonate with findings reported in other international contexts, they should not be interpreted as universally applicable. The ways in which nurses integrate experiential knowledge, relational cues, and emotional appraisal into risk recognition are closely shaped by the organizational structures and sociocultural norms of Chinese obstetric care. Accordingly, the culturally embedded elements identified in this study—such as heightened sensitivity to hierarchical expectations and family involvement—should be understood as context-dependent rather than universal features of clinical judgment. These insights offer valuable perspectives for settings with similar institutional or cultural dynamics, but broader generalization requires caution unless supported by comparative evidence across diverse healthcare systems (45).

In summary, nurses’ performance in obstetric risk recognition is not merely a function of tools and technical knowledge but reflects a complex cognitive process integrating perception, experience, emotion, and reflection. By highlighting how psychological safety, organizational context, and reflective learning interact, this study further extends Tanner’s model to incorporate cyclical, relational, and culturally embedded reasoning processes. It also offers empirical guidance for improving obstetric training systems and advancing institutional strategies for risk governance.

## 5 Conclusion

This study, based on the thematic framework “noticing–interpreting–responding–reflecting,” systematically delineated the mechanisms by which obstetric nurses identify and manage clinical risks in high-stakes environments. These findings revealed that nurses, by establishing a nuanced understanding of the patient’s baseline, are able to detect atypical and contradictory cues with remarkable sensitivity. They skillfully integrate diverse informational sources with clinical experience to balance subjective perceptions and objective indicators, thereby enabling complex health assessments under conditions of uncertainty. In doing so, they demonstrate the capacity for decisive action, prioritization, and team-based collaboration while engaging in continuous reflection and cognitive recalibration—ultimately rendering their risk management more anticipatory and adaptive.

This study addresses a critical gap in the literature dominated by data-driven early warning tools, underscoring the indispensable role of nurses’ subjective judgment and reflective practice. Nurses’ risk sensitivity and capacity for dynamic adjustment constitute a form of tacit knowledge that fortifies the frontline of obstetric safety. Accordingly, there is a pressing need to enhance ongoing nurse training and scenario-based simulation, promote interdisciplinary collaboration and knowledge sharing, and develop institutional support structures that reinforce systematic and precise risk identification. At the same time, greater attention must be given to the psychological wellbeing and professional development of nurses working under pressure to build a maternity care system that is not only safer and more responsive but also emotionally sustainable and professionally empowering.

For nurse educators, hospital administrators, and policymakers, the findings collectively highlight the need for coordinated efforts to translate nurses’ tacit judgment into structured system-level improvements. This entails embedding risk recognition, reflective reasoning, and emotion-aware decision-making into nursing curricula and simulation training; fostering supportive institutional environments that encourage open communication, peer consultation, and non-punitive learning from near misses; and integrating nurses’ experiential insights into maternal safety strategies through adequate staffing, resource investment, and policy frameworks that acknowledge the cognitive and emotional realities of frontline practice. In addition, these actions can transform the tacit competencies identified in this study into sustained, system-wide enhancements in obstetric risk governance.

## 6 Strengths and limitations

This study also has several notable strengths. First, combining reflexive thematic analysis with Tanner’s Clinical Judgment Model provides a strong and innovative analytical structure, allowing the findings to remain both data-driven and theoretically informed. Second, the study applied a comprehensive reflexivity strategy—including analytic memos, researcher dialog, and external audit—which enhances transparency and goes beyond what is typically reported in qualitative studies. Finally, the relatively large sample of 25 obstetric nurses offers substantial variation in experience and clinical settings, strengthening the depth and credibility of the analysis.

This study has several limitations. First, all participants were recruited from tertiary hospitals within a single geographic region, which may limit the generalizability of the findings to other institutional settings or cultural contexts. Second, as with all qualitative interviews, the data are subject to recall and expression bias; certain tacit cognitive processes or emotional responses may not have been fully captured. In addition, the use of individual interviews may have introduced social desirability bias, as participants could have portrayed their decision-making behaviors in ways that align with professional expectations rather than their unfiltered experiences. Third, the interviews relied on retrospective accounts rather than real-time observations of clinical practice, which may have led to partial reconstruction or rationalization of past judgments. Future studies incorporating observational or ethnographic methods could help triangulate the self-reported data and capture nurses’ decision-making as it unfolds in real clinical contexts.

Furthermore, this study did not integrate patient outcome data, which limits the ability to directly link nurses’ risk recognition processes with measurable safety outcomes. Future research should use mixed-method approaches to further investigate the linkages among risk recognition, responsive actions, and patient outcomes. Comparative studies across diverse regions and healthcare systems are also recommended, incorporating perspectives from physicians, midwives, and other professionals to holistically examine the complexity and multidimensional nature of risk recognition in obstetric care.

## Data Availability

The raw data supporting the conclusions of this article will be made available by the authors, without undue reservation.

## References

[1] Chimwaza Y Hunt A Oliveira-Ciabati L Bonnett L Abalos E Cuesta C . Early warning systems for identifying severe maternal outcomes: findings from the WHO global maternal sepsis study. eClinMed. (2024) 79:102981. doi: 10.1016/j.eclinm.2024.102981 PMC11667637 39720608

[2] Gwyneth L. Saving mothers’ lives: the continuing benefits for maternal health from the United Kingdom (UK) confidential enquires into maternal deaths[J]. Semin Perinatol. (2012). doi: 10.1053/j.semperi.2011.09.005 22280861

[3] Baird SM Graves CR. REACT: an interprofessional education and safety program to recognize and manage the compromised obstetric patient. J Perinat Neonatal Nurs. (2015) 29:138–48. doi: 10.1097/JPN.0000000000000098, 25919604

[4] Qiao J Wang Y Li X Jiang F Zhang Y Ma J . A lancet commission on 70 years of women’s reproductive, maternal, newborn, child, and adolescent health in China. Lancet. (2021) 397:2497–536. doi: 10.1016/S0140-6736(20)32708-2, 34043953

[5] Blumenthal EA Hooshvar N McQuade M McNulty J. A validation study of maternal early warning systems: a retrospective cohort study. Am J Perinatol. (2019) 36:1106–14. doi: 10.1055/s-0039-1681097, 30856674 PMC11177629

[6] Smith V Cithambaram K O’Malley D. Early warning systems in maternity care: protocol for a qualitative evidence synthesis of maternity care providers’ views and experiences. HRB Open Res. (2021) 4:59. doi: 10.12688/hrbopenres.13270.1, 35079691 PMC8733824

[7] Hansson T Andersson ME Ahlström G Hansson SR. Women’s experiences of preeclampsia as a condition of uncertainty: a qualitative study. BMC Pregnancy Childbirth. (2022) 22:521. doi: 10.1186/s12884-022-04826-5, 35765045 PMC9241256

[8] Dresser S Teel C Peltzer J. Frontline nurses’ clinical judgment in recognizing, understanding, and responding to patient deterioration: a qualitative study. Int J Nurs Stud. (2023) 139:104436. doi: 10.1016/j.ijnurstu.2023.104436, 36731308

[9] Bernstein SL Catchpole K Kelechi TJ Nemeth LS. Systems-level factors affecting registered nurses during care of women in labor experiencing clinical deterioration. Jt Comm J Qual Patient Saf. (2022) 48:309–18. doi: 10.1016/j.jcjq.2022.02.007, 35370109

[10] Bernstein SL Picciolo M Grills E Catchpole K. A qualitative study of systems-level factors that affect rural obstetric nurses’ work during clinical emergencies. Jt Comm J Qual Patient Saf. (2024) 50:507–15. doi: 10.1016/j.jcjq.2023.12.002, 38220586

[11] Gholipour M Dadashzadeh A Jabarzadeh F Sarbakhsh P. Challenges of clinical decision-making in emergency nursing: an integrative review. Open Nurs J. (2025) 19:51. doi: 10.2174/0118744346378311250320070725

[12] Tanner CA. Thinking like a nurse: a research-based model of clinical judgment in nursing. J Nurs Educ. (2006) 45:204–11. doi: 10.3928/01484834-20060601-04, 16780008

[13] Tanlaka EF Aryal S. Interpretivist constructivism: a valuable approach for qualitative nursing research. Open J Ther Rehabil. (2025) 13:8–19. doi: 10.4236/ojtr.2025.131002

[14] Saunders B Sim J Kingstone T Baker S Waterfield J Bartlam B . Saturation in qualitative research: exploring its conceptualization and operationalization. Qual Quant. (2018) 52:1893–907. doi: 10.1007/s11135-017-0574-8, 29937585 PMC5993836

[15] Braun V Clarke V. One size fits all? What counts as quality practice in (reflexive) thematic analysis? Qual Res Psychol. (2021) 18:328–52. doi: 10.1080/14780887.2020.1769238

[16] Guba EG. Criteria for assessing the trustworthiness of naturalistic inquiries. Ectj. (1981) 29:75–91. doi: 10.1007/BF02766777

[17] Li L Ai C Wang M Chen X. Nurses’ risk perception of adverse events and its influencing factors: a cross-sectional study. INQUIRY J Health Care Organ Provis Financ. (2024) 61:00469580241263876. doi: 10.1177/00469580241263876, 39082075 PMC11292694

[18] Yang M Lin P Zheng L Wu B. Emotional management and clinical communication among nursing students: a single institution experience. Front Psych. (2024) 15:1327629. doi: 10.3389/fpsyt.2024.1327629, 39559280 PMC11570279

[19] Rosa D Villa G Amigoni C Rossetti AM Guberti M Ghirotto L . Role of emotions in the clinical decision-making process of the hospital nurse: a multicentre qualitative study. MethodsX. (2024) 12:102590. doi: 10.1016/j.mex.2024.102590, 38322133 PMC10844854

[20] Zhong Y Hao T Liu X Zhang X Wu Y Wang X . Ethical challenges in information disclosure and decision-making in prenatal testing: a focus group study of Chinese health professionals in maternal and child health services. J Bioethical Inq. (2025) 22:159–73. doi: 10.1007/s11673-024-10376-6, 39162943

[21] Smith SK Benbenek MM Bakker CJ Bockwoldt D. Scoping review: diagnostic reasoning as a component of clinical reasoning in the US primary care nurse practitioner education. J Adv Nurs. (2022) 78:3869–96. doi: 10.1111/jan.15414, 35986584 PMC9805128

[22] Pađen L Pajnič M Vettorazzi R Pérez-Perdomo A Stefaniak M Claes N . “Learning a way of thinking”—world café on clinical reasoning in nursing and midwifery education and practice across five European Union countries. Healthcare. (2023) 11:2969. doi: 10.3390/healthcare11222969, 37998462 PMC10671496

[23] Mousavi Shabestari M Jabbarzadeh Tabrizi F Roshangar F Ghahramanian A Zamanzadeh V Sarbakhsh P . Nurses’ perception of uncertainty in clinical decision-making: a qualitative study. Heliyon. (2024) 10:e36228. doi: 10.1016/j.heliyon.2024.e36228, 39253177 PMC11381593

[24] Shabestari MM Ghahramanian A Roshangar F Tabrizi FJ Zamanzadeh V Sarbakhsh P. Measuring nurses’ uncertainty in clinical decision-making: an integrative review. Open Nurs J. (2023) 17:251. doi: 10.2174/18744346-v17-230727-2023-36

[25] Lavoie P Deschênes M-F Maheu-Cadotte M-A Lapierre A Mailhot T Rodriguez D . Nursing students’ decision-making regarding postpartum hemorrhage: an exploration using the recognition-primed decision model. Nurse Educ Pract. (2022) 64:103448. doi: 10.1016/j.nepr.2022.103448, 36115258

[26] Abdulmohdi N Mcvicar A. Investigating the clinical decision-making of nursing students using high-fidelity simulation, observation and think aloud: a mixed methods research study. J Adv Nurs. (2023) 79:811–24. doi: 10.1111/jan.15507, 36412270 PMC10099619

[27] Reale C Salwei ME Militello LG Weinger MB Burden A Sushereba C . Decision-making during high-risk events: a systematic literature review. J Cogn Eng Decis Mak. (2023) 17:188–212. doi: 10.1177/15553434221147415, 37823061 PMC10564111

[28] Byrne A Massey D Flenady T Connor J Chua WL Lagadec DL. When nurses worry: a concept analysis of intuition in clinical deterioration. J Adv Nurs. (2025) 81:4566–83. doi: 10.1111/jan.16956, 40211551 PMC12271650

[29] Aaron C Miskioglu E Martin KM Shannon B Carberry A. Nurses, managers, and engineers – oh my! Disciplinary perceptions of intuition and its role in expertise development In: 2020 IEEE Frontiers in education conference (FIE). Uppsala, Sweden: IEEE (2020). 1–6. doi: 10.1109/FIE44824.2020.9274026

[30] Andersen HE Toubøl AG. Communities of reflection in nurse education programs: a qualitative multi-methods study. Nurse Educ Today. (2024) 140:106293. doi: 10.1016/j.nedt.2024.106293, 38936042

[31] Rabin S Kika N Lamb D Murphy D AM Stevelink S Williamson V . Moral injuries in healthcare workers: what causes them and what to do about them? J Healthc Leadersh. (2023) 15:153–60. doi: 10.2147/JHL.S396659, 37605753 PMC10440078

[32] Scott Z O’Curry S Mastroyannopoulou K. The impact and experience of debriefing for clinical staff following traumatic events in clinical settings: a systematic review. J Trauma Stress. (2022) 35:278–87. doi: 10.1002/jts.22736, 34672028

[33] Sattar R Lawton R Janes G Elshehaly M Heyhoe J Hague I . A systematic review of workplace triggers of emotions in the healthcare environment, the emotions experienced, and the impact on patient safety. BMC Health Serv Res. (2024) 24:603. doi: 10.1186/s12913-024-11011-1, 38720302 PMC11080227

[34] Ries NM Jansen J. Physicians’ views and experiences of defensive medicine: an international review of empirical research. Health Policy. (2021) 125:634–42. doi: 10.1016/j.healthpol.2021.02.005, 33676778

[35] Taylor C Maben J Jagosh J Carrieri D Briscoe S Klepacz N . Care under pressure 2: a realist synthesis of causes and interventions to mitigate psychological ill health in nurses, midwives and paramedics. BMJ Qual Saf. (2024) 33:bmjqs-2023-016468–538. doi: 10.1136/bmjqs-2023-016468, 38575309 PMC11287552

[36] Ko E Choi Y-J. Debriefing model for psychological safety in nursing simulations: a qualitative study. Int J Environ Res Public Health. (2020) 17:2826. doi: 10.3390/ijerph17082826, 32325983 PMC7215814

[37] Vreugdenhil J Broeksma L Teuwen C Custers E Reinders M Dobber J . Debriefing to nurture clinical reasoning in nursing students: a design-based research study. Nurse Educ Today. (2024) 143:106402. doi: 10.1016/j.nedt.2024.106402, 39278184

[38] Mazzoni D Amadori R Sebri V Tosi M Pregnolato S Surico D . Health anxiety and oppressive support: their impact on decisions for non-urgent use of the emergency department of obstetrics and gynecology. Curr Psychol. (2024) 43:10904–13. doi: 10.1007/s12144-023-05198-5

[39] Raoust GM Bergström J Bolin M Hansson SR. Decision-making during obstetric emergencies: a narrative approach. PLoS One. (2022) 17:e0260277. doi: 10.1371/journal.pone.0260277, 35081113 PMC8791468

[40] Wright E D’Aoust R Swoboda SM Hughes V Hudson K Reller N . Resilience and ethics in nursing education and practice: needs and opportunities. Nurse Educ. (2024) 49:E218–22. doi: 10.1097/NNE.0000000000001580, 38113932

[41] You W Cusack L Donnelly F. Do physicians still direct nursing workforce? A profession striving for autonomy since mid- 1900’s (2022). doi: 10.21203/rs.3.rs-2166336/v1,

[42] McHugh MD Aiken LH Sloane DM Windsor C Douglas C Yates P. Effects of nurse-to-patient ratio legislation on nurse staffing and patient mortality, readmissions, and length of stay: a prospective study in a panel of hospitals. Lancet. (2021) 397:1905–13. doi: 10.1016/S0140-6736(21)00768-6, 33989553 PMC8408834

[43] Wang Y. Patient participation in nursing care in a Chinese hospital: a focused ethnographic study 2025

[44] Nie J-B Smith KL Cong Y Hu L Tucker JD. Medical professionalism in China and the United States: a transcultural interpretation. J Clin Ethics. (2015) 26:48–60. doi: 10.1086/JCE2015261048, 25794294

[45] Pavón Rivera ME Antunez Martinez OF Morales PS. Honduran nurses’ perceptions of advanced nursing care competencies for adults in emergency and critical care: a qualitative study. Int Emerg Nurs. (2025) 81:101640. doi: 10.1016/j.ienj.2025.101640, 40561820

