# Supplementary material for: Uncovering the tacit: a qualitative study of obstetric nurses’ risk recognition and decision-making in perinatal care
Source: Front Med (Lausanne). 2025 Dec 19;12:1701924. doi: 10.3389/fmed.2025.1701924 (PMC12758021; doi:10.3389/fmed.2025.1701924)
Supplement: Supplementary file 1 [file Supplementary_file_1.docx]

| Phase (Tanner’s Model) | Interview Questions (13 total) |
| --- | --- |
| Noticing | 1. In your daily work, how do you determine what is “normal” for each woman under your care? |
|  | 2. How do you recognize subtle but unusual changes in a postpartum woman’s condition? |
|  | 3. Have you ever noticed atypical cues—such as changes in tone, mood, or behavior—that made you sense potential risk even when clinical indicators appeared normal? |
|  | 4. How does your previous clinical experience or intuition influence what draws your attention first? |
| Interpreting | 5. When you detect a change, how do you interpret or make sense of it? |
|  | 6. Have you ever faced conflicting information (for example, when patient complaints and objective indicators did not match)? How did you handle that? |
|  | 7. What types of information do you usually integrate—such as vital signs, patient communication, team input, and emotional cues—when forming your judgment about risk? |
| Responding | 8. When clinical indicators appear normal but you sense potential risk, what actions do you typically take? |
|  | 9. How do you decide which issue to prioritize when multiple problems arise at the same time? |
|  | 10. How do you communicate your concerns or uncertainty with doctors or colleagues? |
|  | 11. Can you recall a situation where you felt compelled to act quickly despite incomplete evidence? What guided your decision? |
| Reflecting | 12. Have you ever repeatedly reflected on a clinical decision that may have been a misjudgment and found it affected your later practice? |
|  | 13. How have your past experiences, near misses, or emotional reactions shaped your vigilance and approach to clinical risk today? |
