# Supplementary material for: Uncovering the tacit: a qualitative study of obstetric nurses’ risk recognition and decision-making in perinatal care
Source: Front Med (Lausanne). 2025 Dec 19;12:1701924. doi: 10.3389/fmed.2025.1701924 (PMC12758021; doi:10.3389/fmed.2025.1701924)
Supplement: Supplementary file 2 [file Table_1.docx]

| Table 1. Thematic Framework of Obstetric Nurses' Risk Recognition Across Tanner’s Clinical Judgment Model, With Representative Quotes | | |
| --- | --- | --- |
| Phase (Tanner’s Model) | Subthemes | Representative Quotes |
| Noticing | Recognizing baseline conditions | “She’s usually very active, likes walking around and chatting with others. ”（N20） |
|  | Detecting atypical cues | “Her voice became faint, she avoided eye contact, and appeared exhausted—this was not her normal demeanor. ”（N19） |
|  | Experience-driven vigilance | “Based on my experience with mastitis cases, when I saw her frown during breastfeeding and noticed localized redness, I immediately recommended a breast exam. ”（N11） |
| Interpreting | Synthesizing multiple cues | “Her blood pressure rose slightly to 140/90 mmHg, and she kept rubbing her temples and said her vision was blurry. Considering her history of gestational hypertension and that it was day three postpartum, I suspected postpartum hypertension syndrome. ”（N13） |
|  | Conflicting Signals and Dynamic Balancing | “She repeatedly told me she felt like she was going to collapse, but all her vitals were within range. After talking to her family, I suspected it was a stress response. ”（N16） |
| Responding | Prioritizing action | “The patient suddenly experienced heavy vaginal bleeding and a sharp drop in blood pressure. I immediately established an IV line for rapid fluid resuscitation, called the physician, and prepared emergency medications to stabilize her vital signs. ”（N15） |
|  | Communication and Peer Confirmation | “I reported the situation to the on-duty doctor and discussed it with a senior nurse. ”（N10） |
|  | Pressure Under Uncertainty | “Sometimes we’re really not sure—like when a slight fever might or might not mean an infection. If we report it, it leads to lab tests, hospitalization, and stress for the family. But if we don’t, and something goes wrong, I’d be consumed with guilt. I spend the whole night worrying. ”（N2） |
| Reflecting | Recurrent Self-Review and Doubt | “I once managed a case of postpartum hemorrhage. Although the patient stabilized in the end, I still wonder whether I should’ve responded faster or called the doctor earlier. It leaves me uneasy. ”（N9） |
|  | Reflective Practice | “After managing a case of postpartum depression, I realized I had been too focused on physiological indicators and overlooked the patient’s emotional cues. ”（N1） |
|  | Heightened Risk Sensitivity | “Since I handled a case of severe postpartum hemorrhage, I’ve become hypervigilant. If a patient shows even slight discomfort, I immediately order a blood test. Sometimes results are normal, but I’d rather be safe than sorry. ”（N13） |
